# SARS-CoV-2 Wastewater Surveillance for Public Health Action

**DOI:** 10.3201/eid2709.210753

**Published:** 2021-09

**Authors:** Jill S. McClary-Gutierrez, Mia C. Mattioli, Perrine Marcenac, Andrea I. Silverman, Alexandria B. Boehm, Kyle Bibby, Michael Balliet, Francis L. de los Reyes, Daniel Gerrity, John F. Griffith, Patricia A. Holden, Dimitrios Katehis, Greg Kester, Nathan LaCross, Erin K. Lipp, Jonathan Meiman, Rachel T. Noble, Dominique Brossard, Sandra L. McLellan

**Affiliations:** University of Wisconsin–Milwaukee School of Freshwater Sciences, Milwaukee, Wisconsin, USA (J.S. McClary-Gutierrez, S.L. McLellan);; Centers for Disease Control and Prevention, Atlanta, Georgia, USA (M.C. Mattioli, P. Marcenac);; University Tandon School of Engineering, Department of Civil and Urban Engineering, Brooklyn, New York, USA (A.I. Silverman);; Stanford University Department of Civil and Environmental Engineering, Stanford, California, USA (A.B. Boehm);; University of Notre Dame Department of Civil and Environmental Engineering and Earth Sciences, Notre Dame, Indiana, USA (K. Bibby);; County of Santa Clara Department of Environmental Health, San Jose, California, USA (M. Balliet);; North Carolina State University Department of Civil, Construction, and Environmental Engineering, Raleigh, North Carolina, USA (F.L. de los Reyes III);; Southern Nevada Water Authority Applied Research and Development Center, Las Vegas, Nevada, USA (D. Gerrity);; Southern California Coastal Water Research Project, Costa Mesa, California, USA (J.F. Griffith);; University of California Bren School of Environmental Science and Management, Santa Barbara, California, USA (P.A. Holden);; New York City Department of Environmental Protection, New York, New York, USA (D. Katehis);; California Association of Sanitation Agencies, Sacramento, California, USA (G. Kester);; Utah Department of Health, Salt Lake City, Utah, USA (N. LaCross);; University of Georgia Department of Environmental Health Science, Athens, Georgia, USA (E.K. Lipp);; Wisconsin Department of Health Services, Madison, Wisconsin, USA (J. Meiman);; University of North Carolina at Chapel Hill Institute of Marine Sciences, Morehead City, North Carolina, USA (R.T. Noble);; University of Wisconsin–Madison Department of Life Sciences Communication, Madison (D. Brossard);; University of Wisconsin–Madison Morgridge Institute for Research, Madison (D. Brossard)

**Keywords:** COVID-19, coronavirus disease, SARS-CoV-2, severe acute respiratory syndrome coronavirus 2, viruses, respiratory infections, zoonoses, SARS-CoV-2 wastewater surveillance

## Abstract

Wastewater surveillance for severe acute respiratory syndrome coronavirus 2 (SARS-CoV-2) has garnered extensive public attention during the coronavirus disease pandemic as a proposed complement to existing disease surveillance systems. Over the past year, methods for detection and quantification of SARS-CoV-2 viral RNA in untreated sewage have advanced, and concentrations in wastewater have been shown to correlate with trends in reported cases. Despite the promise of wastewater surveillance, for these measurements to translate into useful public health tools, bridging the communication and knowledge gaps between researchers and public health responders is needed. We describe the key uses, barriers, and applicability of SARS-CoV-2 wastewater surveillance for supporting public health decisions and actions, including establishing ethics consideration for monitoring. Although wastewater surveillance to assess community infections is not a new idea, the coronavirus disease pandemic might be the initiating event to make this emerging public health tool a sustainable nationwide surveillance system, provided that these barriers are addressed.

Wastewater surveillance for severe acute respiratory syndrome coronavirus 2 (SARS-CoV-2) is rapidly evolving as a public health tool that holds both promise and challenges (*1*–*3*). In concept, a sewer system contains biological waste from the human population it serves. Biological constituents, including pathogens, enter the sewer system through feces, urine, saliva, and other excreta, and the pathogen concentrations represent input from the human population served by the network of pipes within the sewershed. Given that SARS-CoV-2 RNA is shed in feces of persons with asymptomatic and symptomatic infections (*4*,*5*), the potential for coronavirus disease (COVID-19) community-level surveillance through wastewater has garnered much attention since the first report of detection of SARS-CoV-2 RNA in wastewater in March 2020 (*6*).

SARS-CoV-2 wastewater surveillance could be an important complement to existing public health surveillance for the COVID-19 response because it has the ability to provide information on infection trends in newly reported cases in a community without being influenced by availability of and access to clinical testing resources or data on healthcare-seeking behavior (*1*,*2*,*7*). However, practical and technologic challenges to implementing and interpreting this new surveillance tool need to be addressed. Precisely measuring levels of virus in a complicated wastewater matrix requires specialized equipment and expertise, and quality-control and quality-assurance procedures distinct from clinical testing are necessary for precise molecular quantification (*8*).

During the pandemic, the science and engineering research communities and commercial laboratories have made a tremendous effort to develop SARS-CoV-2 RNA detection and quantification methods for wastewater surveillance (*9*–*11*). As a result of these concerted efforts, SARS-CoV-2 RNA concentrations are now being measured in many wastewater systems globally, and the data are showing wastewater viral RNA concentration trends are correlated with trends in new cases reported days to weeks later, depending on reporting lags (*6*,*12*–*14*). Some public health managers are already integrating these data into their COVID-19 response decision-making processes (L.B. Stadler et al., unpub. data, ).

Despite the technologic advances, barriers remain to using wastewater surveillance data to inform public health decisions. Of note, there is a communication gap between the laboratories that quantify SARS-CoV-2 RNA in wastewater and the public health practitioners tasked with incorporating wastewater data into existing surveillance frameworks, which includes reported COVID-19 cases, hospitalizations, and deaths. Bridging the gap between research groups generating wastewater surveillance data and the public health sector might help to harness the long-term potential of SARS-CoV-2 wastewater surveillance as a tool for public health disease surveillance and decision-making.

In an effort to bridge this identified gap, the Sloan Foundation supported a group of academic investigators to convene an interdisciplinary expert group with the objective of facilitating conversations around the opportunities, limitations, and challenges in using SARS-CoV-2 wastewater data in public health action. The perspective we will describe was formed from a group of environmental microbiology, engineering, wastewater, and public health experts, as well as from opinions shared during 3 focus group discussions with officials from 10 state and local public health agencies. Common definitions of wastewater surveillance terminology are provided in the Appendix .

## Interdisciplinary Focus Group Discussions

An interdisciplinary group of experts in environmental virology, environmental microbiology, wastewater engineering, and public health was brought together by authors S.L.M., A.B.B., A.I.S., K.B., and D.B. to discuss barriers, best practices, and data use by public health and to develop this article. The group, consisting of the authors, represented experts from 10 academic or research institutions, 2 wastewater agencies, 1 city environmental department, and public health practitioners from 1 county and 2 state health departments that had already begun to develop or implement wastewater surveillance programs in their jurisdictions as part of their COVID-19 response. Together, this group represented a cross section of US institutions involved in SARS-CoV-2 wastewater research. Expertise in life science communication was also represented. The research members of the group met weekly or biweekly from July 2020 through September 2020 to discuss technical aspects of wastewater surveillance, followed by meetings of the entire group to discuss barriers and data use by public health entities.

In November 2020, this interdisciplinary expert group further convened 3 focus groups to better understand current perspectives of public health responders on the barriers to using SARS-CoV-2 wastewater surveillance data and how wastewater data could support local public health decisions during the rapidly evolving pandemic. We recruited participants through the authors’ professional networks or from suggestions of those who were initially contacted. Divided into 3 separate virtual meetings, the focus groups included expert group members; officials from 2 additional wastewater utilities; and officials from 1 city, 1 district, 3 county, and 3 state public health departments from urban and rural communities. Moreover, epidemiologic and laboratory lead staff from the Centers for Disease Control and Prevention’s National Wastewater Surveillance System (NWSS; National Center for Emerging and Zoonotic Infectious Diseases, Division of Foodborne, Waterborne, and Environmental Diseases) participated in the focus groups. Focus group participants were provided with premeeting materials that posed questions on 3 general areas: current use and expectations of wastewater surveillance for SARS-CoV-2; 2 concerns, questions, and confidence surrounding the tool; and long-term applications. Because of the sensitivity of response-related data and resulting public health action for COVID-19, focus groups were not recorded to enable open discussion of data interpretation and challenges. Expert group members summarized and reviewed attendee responses without the use of analytical software. The University of Wisconsin–Milwaukee Institutional Review Board review of this project and granted it Category 2 exempt status (approval no. 21.132). We obtained informed consent from all focus group attendees.

On the basis of the results of the expert group discussions and the focus groups, we determined the major barriers identified by public health officials for implementing and using data from wastewater-based infectious disease surveillance programs (Table 1). We also highlight methodologic best practices for wastewater researchers and testers to facilitate use of wastewater data by public health officials. Finally, we point toward critical actions needed by both wastewater surveillance method developers and program implementers to effectively incorporate wastewater surveillance into the COVID-19 public health response. 

**Table 1:** Summary of barriers, best practices, and future needs for public health agencies using wastewater surveillance data for public health action

Barrier	Recommended best practices	Future needs (key strategy areas)
Many public health agencies are not yet comfortable interpreting wastewater data	• Communicate results interpretation alongside data limitations and known variability sources • Collaborate with laboratories, wastewater utilities, environmental health departments, and communications experts	Evaluation of wastewater data variability and uncertainty sources in a variety of systems (research)
Public health agencies want to see wastewater data in their own communities to gain confidence in utility	• Provide case studies from community applications and perspectives • Perform retrospective analyses on existing datasets	Documentation of wastewater surveillance use cases for adoption in different communities and infrastructure systems (research and communication)
New knowledge and investment needed to sustain wastewater surveillance systems	• Co-develop programs and methods with scientific experts and government agencies • Share methods and experiences across research, wastewater, and public health	Investment in physical laboratory capacity, personnel, and interagency collaboration frameworks (organizational structures and policy)
Ethics of wastewater surveillance data sharing and use not yet established	• Evaluate sample anonymity • Engage the public in collection and data use	Development of ethical wastewater data use standards for surveillance and research (policy and research)

### Barrier 1: Wastewater Surveillance as New Data Source 

As a new data source, most public health agencies are not yet comfortable interpreting wastewater data. During focus group discussions, over half of public health representative focus group participants highlighted in their discussion that personnel and resources are stretched well past capacity, resulting in a limited ability to incorporate new and unfamiliar metrics into the workload, especially without demonstration of their value in decision making. Unlike case counts or hospitalizations that have a relationship to disease in the community, wastewater surveillance data are presented as concentrations of SARS-CoV-2 gene copies per volume of wastewater (commonly expressed as per liter of sewage or per gram of solids), which might be difficult for persons unfamiliar with the measurement to contextualize, leading to challenges in interpreting the data and results. Reporting wastewater data can be even further complicated because, to compare across time and space, the wastewater data are often normalized by total daily wastewater flow (expressed as SARS-CoV-2 gene copies per day) or by the concentration of a human-specific gut microbe (*15*). Our focus groups identified several additional reasons for the hesitation in using wastewater data for public health responses, which can be grouped into 2 main categories.

#### Uncharacterized Sources of Uncertainty and Variability

Many factors can influence SARS-CoV-2 RNA concentrations measured in wastewater, such as sampling location, sampling methods (e.g., grab versus flow-weighted composite samples), sewer transit time, addition of industrial waste or stormwater to the sewer, wastewater flow rates, and fecal shedding rates. These factors are being investigated in research studies, and their effects on SARS-CoV-2 RNA concentrations are still being defined; therefore, the degree of natural variability and acceptable uncertainty in the data are not fully known for wastewater surveillance. A recent meta-analysis of published reports summarized and quantified the variability associated with several factors, including fecal shedding, sewer transit, sampling, storage, and analyses (*16*).

#### Lack of Methodologic Standardization

Public health laboratories are accustomed to testing samples by using highly standardized methods with defined levels of uncertainty. The variability in SARS-CoV-2 RNA wastewater measurements introduced when concentrating the virus from a large volume or during RNA extraction or RNA quantification is still being investigated as part of methodologic evaluations (*17*–*19*). In addition, no single standard method exists for concentrating and measuring SARS-CoV-2 RNA from wastewater. In fact, a single method might not be appropriate for all wastewater sources, because wastewater composition varies across locations or for all phases in the epidemic, given the differing applications and data needs (*20*). For example, concentration of viral RNA might not have been needed at some locations during times of high COVID-19 prevalence but is likely needed at times when prevalence is low and fewer persons are shedding viruses. Each method might be associated with different levels of uncertainty and variability that must be defined using appropriate preanalytical and analytical method controls and replication.

Delineating sources of data uncertainty, defining variability in measurement, and standardizing methods represent important avenues of inquiry for research (*2*). Further, more information is needed on the rate of fecal shedding of infected persons (both symptomatic and asymptomatic) during the course of disease or carriage. In the meantime, strategies exist that can be adopted for communicating results across the many different entities that are generating and evaluating SARS-CoV-2 wastewater data. First, results should be coupled with explanations of data limitations and known sources of variability, which can help assimilate the results into decision-making as public health agencies become more accustomed to the data. In addition, close collaborations between groups generating wastewater data and public health agencies, wastewater utilities, and experts in communication and data visualization are needed to ensure that findings are appropriately communicated to data end-users to prevent false assumptions and under-interpretation or overinterpretation. Environmental health departments might be good liaisons between different wastewater surveillance partners because, even though they might not be organized within the public health department, they often have both extensive public health and wastewater knowledge.

### Barrier 2: Application and Utility of Wastewater Surveillance in Communities

Public health agencies want to see SARS-CoV-2 wastewater data in their own communities to gain confidence in its application and utility at different scales and under different scenarios. Multiple applications for wastewater surveillance exist, and each has a complex set of considerations and limitations that will affect practitioners’ confidence for using data in public health decisions. In addition, every community has unique infrastructure, demographics, and public health capacity and challenges that will inevitably influence how measurements of SARS-CoV-2 RNA in wastewater can be used. Public health agencies beginning wastewater surveillance programs should consider the type of information that would be most useful for their response needs and design sampling at the appropriate scale. When asked what would increase confidence in using wastewater surveillance data, several focus group participants reported substantial benefit from seeing the data in action in their own communities, enabling them to gain a greater understanding of the data and its potential value. Wastewater data can be collected at 3 different scales (*21*), and the experts group identified considerations for each scale.

#### Wastewater Treatment Plant

Wastewater sampling routinely occurs at wastewater treatment plants for permit compliance requirements, so additional sampling at the plant is usually straightforward to implement. Wastewater treatment plants can serve sewersheds containing thousands to millions of people, depending on their size, and measurements of SARS-CoV-2 RNA in wastewater collected at the plant can provide insight into infection burdens in the sewershed population. Several cities are now reporting correlations between SARS-CoV-2 concentrations at wastewater treatment plants and diagnosed COVID-19 cases in the sewershed service area (*7*,*9*,*14*,*22*–*24*).

#### Subsewershed

Depending on the data needs of a wastewater surveillance effort, sampling smaller geographic areas within a community might be needed. Wastewater can be samples from the pipe network that moves waste from households and businesses to the wastewater plant, thus isolating a subsewershed population. Collecting samples from within the pipe network is complicated by various factors, including lack of adequate maps and challenging access to manholes. Depending on the wastewater infrastructure design and equipment resources, sampling at the subsewershed scale can be resource-intensive, and appropriate sampling schemes for the approach presently lack validation.

#### Facility-level

Information on COVID-19 infections of persons working and living in individual facilities can potentially be obtained by testing wastewater from the facilities (e.g., hospitals, skilled nursing facilities, schools, or universities) (*25*). Drawings of facilities’ plumbing will be necessary to identify potential sampling locations, and intermittent use of water within the facilities will result in intermittent flow in the plumbing, which can challenge sampling efforts (*26*). Given the smaller population being sampled, wastewater testing might give a false-negative result when cases are present because of difficulty in obtaining a representative sample, inconsistent (or absent) viral shedding in feces by infected persons, or low sensitivity in the method (J. Crowe et al., unpub. data, ). Although evidence exists of wastewater testing being implemented at the building-level at >200 universities globally (C.C. Naughton et al., unpub. data, ), the details of only a few are available in the literature. Available case studies demonstrate differing usage of wastewater data; some evaluated wastewater data as a confirmatory measure alongside clinical surveillance testing (*27*; K. Reeves et al., unpub. data, ) and others used wastewater detections to trigger surge testing at specific residences or campus-wide (*26*,*28*; S.A. Travis et al., unpub. data, https://doi.org/10.1101/2021.03.02.21252746). An analysis of programs at 25 universities revealed that, in addition to technical feasibility, consideration of interpretation and communication of data and follow-up actions were important (*29*). The most successful uses appear to have integrated wastewater testing with clinical testing and contact-tracing responses; however, as more information becomes available, critically evaluating these applications for their long-term utility and cost-effectiveness will be important. In some cases, routine screening of persons might allow for more immediate isolation of cases and contract-tracing.

We should note that across the United States, 80% of the population is served by a piped sewage network, whereas the remaining use cesspools or septic systems (*30*). Little evidence exists supporting the utility of sewage surveillance in these onsite sanitation systems.

Although the importance of seeing the application of wastewater SARS-CoV-2 data in public health practitioners’ own communities cannot be overstated, providing clear case examples of other community applications and perspectives across different jurisdictions and areas is recommended to improve confidence in these novel surveillance data. As the field of wastewater surveillance advances, a growing body of literature describes examples of use cases (*9*,*22*,*24*,*31*). In addition, retrospective analyses and peer-reviewed reporting of case studies should be encouraged as a means of increasing decision-making confidence for future related scenarios. Some communities have been generating wastewater SARS-CoV-2 datasets since early in the pandemic, giving them the ability to perform retrospective analysis to demonstrate whether SARS-CoV-2 wastewater data effectively captured reported case trends, filled gaps in case trends in areas with more limited clinical testing, or both. Public health implementers from the expert group provided specific examples of how wastewater data were used to support their COVID-19 response (Table 2; Appendix).

**Table 2:** Examples of how SARS-CoV-2 wastewater data have been used by public health departments to support their COVID-19 response, United States*

Location	Description
Santa Clara County, California	The County of Santa Clara Emergency Operations Center and Public Health Department engaged in early evaluation of wastewater surveillance for SARS-CoV-2 in partnership with Stanford University researchers. A monitoring approach was developed to analyze SARS-CoV-2 RNA in settled solids at all 4 wastewater treatment plants in the county, accounting for >95% of the county’s total estimated population of 2 million. The county has observed trends in measured SARS-CoV-2 RNA from solids to generally track with positive COVID-19 case data. Evaluation is ongoing to understand what public health actions might be implemented in response.
Utah	Utah’s SARS-CoV-2 wastewater monitoring program began with in March 2020 as a collaboration between the Utah Department of Environmental Quality, Utah Department of Health, and 4 academic laboratories, which extended to wastewater facilities statewide by July 2020. The wastewater surveillance data have been used to help direct mobile testing teams to areas with low prevalence of clinical testing, determine where to send mask-wearing compliance observers, and assist the interpretation of other surveillance data. Consistently decreasing SARS-CoV-2 RNA concentrations in wastewater supported the conclusion that the observed declining case rates were real. Utah developed a public dashboard (https://wastewatervirus.utah.gov).
Wisconsin	The Wisconsin Department of Health Services initiated a statewide SARS-CoV-2 wastewater testing program in collaboration with the Wisconsin State Laboratory of Hygiene and the University of Wisconsin–Milwaukee. This program has monitored SARS-CoV-2 RNA concentrations in samples collected from 70 municipal wastewater treatment plants that cover 50% of the state population. Sample collection for select locations began in August 2020 and captured the pre-Thanksgiving surge in COVID-19 cases in northeastern Wisconsin. Local health departments have used these data to confirm health trends identified through clinical testing, particularly in rural areas of the state with limited testing access. Data are publicly available (https://www.dhs.wisconsin.gov/covid-19/wastewater.htm).

*Examples were provided by public health practitioners from the expert group. A detailed description of these activities is provided in the Appendix. COVID-19, coronavirus disease; SARS-CoV-2, severe acute respiratory syndrome coronavirus 2.

### Barrier 3: Lack of Institutional Knowledge and Resources

New institutional knowledge, organizational leadership, and investment in resources and personnel are needed to sustain wastewater surveillance systems. Current efforts to monitor wastewater for SARS-CoV-2 have developed in an ad hoc manner during an active pandemic. The environmental virology equipment required for sample processing are not typically available in public health or wastewater laboratories. Thus, many research laboratories initially conducted the laboratory analysis for current surveillance programs. However, this approach will likely not be sustainable and instead necessitates transfer of these functions to municipal, public health, or commercial laboratories.

Research laboratories and public health agencies that were early adopters of this technology can assist in this transition by partnering with local laboratories and promoting data and methods sharing across the research, wastewater, and public health sectors. Transferring technical knowledge between researchers and laboratories implementing these methods will ideally occur early during program implementation. As an example, in establishing their SARS-CoV-2 wastewater monitoring program, the New York City (NYC) Department of Environmental Protection engaged academic partners at New York University, Queens College, and Queensborough Community College (both in New York, New York, USA. All methodologic development work for this program occurred in NYC Department of Environmental Protection’s own laboratory, with academic partners and NYC laboratory analysts working side-by-side in methodologic optimization and implementation. This collaboration enabled multidirectional workforce capacity building and exchange of technical information, ultimately resulting in an ongoing and self-sufficient wastewater monitoring program in NYC. These types of stakeholder relationships can aid widespread implementation in the county and state.

Early in the pandemic, in many municipalities, no organizational structure or identified agency existed to facilitate wastewater surveillance implementation. Because of the multidisciplinary nature of wastewater surveillance, multiple partners have a critical role. However, active working relationships across wastewater and public health agencies rarely exist. Although municipal wastewater agencies are actively engaged in public health disease prevention by treating wastewater, they often are not engaged in infectious disease response efforts. Central to our discussions with public health and wastewater practitioners was an overwhelming desire for an improved organizational structure between the various stakeholders needed to conduct a wastewater surveillance program. In particular, new organizational leadership is needed to improve the efficiency of wastewater surveillance program implementation.

In response to the COVID-19 pandemic, researchers partnered with wastewater and public health agencies to launch current wastewater surveillance efforts. Because of the many stakeholders involved in these efforts, co-development of programs and methods across various experts and agencies is needed to ensure efficient and successful development. Furthermore, as research continues to advance, sharing of methods and experiences between researchers and new partners in infectious disease response can identify needs, enable knowledge transfer, and build longer term relationships to promote partner-driven research. Investments in physical laboratory capacity, personnel, and interagency collaboration frameworks to build this new institutional knowledge into public health surveillance frameworks for future epidemics can ensure that these partnerships are valuable in the long term. The Centers for Disease Control and Prevention is taking a leadership role by forming the NWSS and developing national data reporting standards and analytics systems, as well as supporting state, local, and territorial capacity building necessary to ensure a sustainable and efficient public health surveillance system (*32*) (Appendix). 

### Barrier 4: Ethics Considerations

The ethics of wastewater surveillance data collection, sharing, and use are not yet established. Wastewater SARS-CoV-2 RNA concentration data collected in appropriately large sewersheds are not individually identifiable, but concerns over stigma or privacy might occur if collecting samples from a sufficiently small population or specific community, when persons might be identified through deductive disclosure (*2*,*20*). Some public health agencies and wastewater utilities are therefore hesitant to engage with wastewater surveillance data because of a lack of clarity over privacy, confidentiality, regulatory, and ethics issues and concerns. Within our own focus groups, one third of participants voiced these concerns. Public health agencies are entrusted to protect the broader public and therefore must ensure that their efforts are not inadvertently leaving out or inappropriately targeting certain demographic groups because of infrastructure access or design constraints. In contrast to healthcare data, environmental monitoring data are typically not considered a protected data type, and this disconnect represents an additional challenge to integrating wastewater data into public health data streams. As genomic sequencing approaches are applied to wastewater surveillance to evaluate emerging SARS-CoV-2 variants (*33*), methods that inventory the total genetic signal, such as metagenomics, also have the potential to contain identifiable personal genetic information. Data reporting standards could require exclusion of human genetic information and wastewater sample location information.

Previous applications of wastewater surveillance for evaluating illicit drug use or poliovirus circulation have raised similar data concerns, primarily determining that samples should be collected from sufficiently large populations to ensure sample anonymity (*34*). However, adopting wastewater surveillance for SARS-CoV-2 has occurred under unique emergency circumstances where higher resolution surveillance data was critical to the response, leading to new ethics challenges that have not been previously considered or resolved. Efforts are underway by both the research and governmental communities to evaluate the ethics and privacy limitations for wastewater surveillance data. As these efforts continue, researchers and practitioners should consider ethics use of wastewater surveillance data by evaluating sample anonymity on a case-by-case basis and engaging the public in sample collection and data use efforts. Although ensuring the ethical use of these data is paramount, wastewater surveillance data might be uniquely able to address some of the inadvertent biases of other public health surveillance systems that depend on healthcare access and health-seeking behaviors.

## Conclusions

SARS-CoV-2 wastewater data have added value as a biologically independent, passive source of data that public health agencies can take advantage of for the COVID-19 pandemic response. As research on wastewater testing for SARS-CoV-2 continues, the methods used to generate and analyze these data are evolving and are undergoing rigorous evaluation, which will reduce the uncertainties associated with this new data source. For widespread adoption as a public health tool, 2-way communication and knowledge co-development might ensure that wastewater data have clear value in addressing public health needs, are simple to integrate into other surveillance and health systems, and are used for public health decisions and actions. The field of wastewater surveillance is rapidly evolving, and continued reporting of use cases in the peer-reviewed literature will play an important role in validating this approach.

As the pandemic moves to a new phase because of vaccine availability, wastewater surveillance might be useful for identifying areas in a community where SARS-CoV-2 viral shedding is not declining and thus could be targeted for increased vaccination efforts (*35*). Further, many wastewater surveillance programs are shifting focus to tracking variants through wastewater (*33*,*36*,*37*) to complement sequencing clinical samples. The COVID-19 pandemic might be the motivating event for creating a sustainable structure to support wastewater surveillance as a unique approach for community-level health monitoring purposes. Investments in resources and personnel can create and sustain a robust wastewater surveillance system for public health emergencies and maintain relationships among stakeholders involved in wastewater surveillance programs. Such investments will continue to build institutional knowledge to support the integration of wastewater data into surveillance frameworks for public health actions.

Appendix Additional information about SARS-CoV-2 wastewater surveillance for public health action.
