# Supplementary material for: SARS-CoV-2 Wastewater Surveillance for Public Health Action
Source: Emerg Infect Dis. 2021 Sep;27(9):e210753. doi: 10.3201/eid2709.210753 (PMC8386792; doi:10.3201/eid2709.210753)
Supplement: Appendix — Additional information about SARS-CoV-2 wastewater surveillance for public health action. [file 21-0753-Techapp-s1.pdf]

Article DOI: https://doi.org/10.3201/eid2709.210753

# SARS-CoV-2 Wastewater Surveillance for Public Health Action

## Appendix

## Key Wastewater Surveillance Terms

#### Grab samples

Samples collected as a single “grab” volume at a single point in time

#### Composite samples

Samples collected by pooling multiple grab samples over a set time period. Flow-weighted composite samples are pooled after set flow intervals (e.g., one sub-sample per 200,000 gallons of flow); composite samplers refer to instruments used to automatically collect and pool sample volumes at specific intervals to create a composite sample

#### Sewer transit time

The average time for sewage to travel from an upstream source (e.g., toilet flush) to a downstream sampling point (e.g., treatment plant)

#### Solids

The nonaqueous fraction of sewage, which may be in the untreated sewage sample or accumulated during the treatment process

#### Method controls

A range of additional measurements needed to ensure method integrity and appropriate interpretation of SARS-CoV-2 RNA concentration data, including a matrix recovery control, human fecal normalization, quantitative measurement controls, and controls to assess molecular method inhibition

#### Replication

The same procedure performed multiple times to assess precision of the measurement

### Uncertainty

Uncertainty can refer to unknown relationships between a measurement and another metric, such as diagnosed cases. Uncertainty can also be introduced because of variability in measurements due to representative sampling, technical precision, or instrument error.

## Examples of How Wastewater Data Were Used by Public Health Implementers to Support Their COVID-19 Response

### Wisconsin

The Wisconsin Department of Health Services (WI DHS) initiated a statewide SARS-CoV-2 wastewater testing program in collaboration with the Wisconsin State Laboratory of Hygiene and the University of Wisconsin-Milwaukee. To date, this program has monitored SARS-CoV-2 RNA concentrations once or twice per week in samples collected from 70 municipal wastewater treatment plants that provide service for ≈53% of the state's population. Sample collection for select locations began in August 2020 and captured the pre-Thanksgiving surge in COVID-19 cases in northeastern Wisconsin. By including a large number of wastewater treatment plants of various sizes in the program, WI DHS is able to assess correlations between SARS-CoV-2 concentrations in wastewater and diagnosed COVID-19 cases and hospitalizations and identify factors that influence these relationships. Data are publicly available (https://www.dhs.wisconsin.gov/covid-19/wastewater.htm). Local health departments have used these data to confirm health trends identified through clinical testing, particularly in rural areas of the state with limited testing access. A short turnaround time for wastewater analysis will allow WI public health officials to identify regions with increasing SARS-CoV-2 transmission and anticipate surges in COVID-19 hospitalizations. (Example provided by J. Meiman)

### Utah

Utah's SARS-CoV-2 wastewater monitoring program began with a limited pilot project in March 2020 as a collaboration between the Utah Department of Environmental Quality (UDEQ), Utah Department of Health (UDOH), and four academic laboratories. Upon successful completion of the pilot, sampling was extended in July 2020 to wastewater facilities statewide. As of January 2021, the program collects samples twice a week from 33 facilities that serve

≈87% of the state's population. Utah developed a public dashboard (wastewatervirus.utah.gov), integrated the data into a restricted access UDOH internal dashboard, and currently disseminates a summary of new data several times a week to local health departments, UDOH leadership, and other pandemic response personnel. To date, wastewater surveillance data have been used to help direct clinical testing resources (particularly mobile testing teams) to areas with low prevalence of clinical testing, determine where to send mask-wearing compliance observers, and assist the interpretation of other surveillance data. As an example, in July 2020, the wastewater surveillance data indicated declining case rates in some regions of the state. However, the number of people being tested was also decreasing in some of these areas, raising the possibility that the declining case rates were artifacts of clinical testing efforts. Consistently decreasing SARS-CoV-2 RNA concentrations in wastewater were able to support the conclusion that the observed declining case rates were real. Wastewater data were a leading indicator for reported cases, with sewershed-associated case rates showing trends 4–7 days after wastewater levels. (Example provided by N. LaCross).

**Santa Clara County, California**

The County of Santa Clara Emergency Operations Center and Public Health Department serve a population of almost two million residents and engaged in early evaluation of wastewater surveillance for SARS-CoV-2 in partnership with Stanford University researchers. Using a multidisciplinary team, a monitoring approach was developed to analyze SARS-CoV-2 RNA in settled solids at all four wastewater treatment plants in the County, comprising over 95% of the County's total population. A pilot project involving four regional wastewater treatment plants provided daily measurements with a 24-hour turnaround time. This fast turnaround allows County officials to see trends in wastewater data before receipt of clinical data because of lags in the reporting of clinical test results (5–14 days in late 2020 through early 2021). The County and their partners continue to evaluate wastewater surveillance data in conjunction with other public health data to better understand the COVID-19 trends as well as limitations in interpretation of wastewater data. As an example, the County has observed trends in measured SARS-CoV-2 RNA from the wastewater surveillance to generally track with positive COVID-19 case data in the four sewersheds being evaluated. The County will continue to evaluate the data over the next several months to determine if additional trends can be identified and to understand what public health actions might be implemented in response. (Example provided by M. Balliet)

## CDC National Wastewater Surveillance System

CDC NWSS was launched to provide the infrastructure to overcome many of these barriers in the U.S., as it provides a robust, highly adaptable system for community-level disease surveillance that can be expanded to collect data on multiple pathogens, such as antibiotic resistant bacteria and enteric pathogens, and leveraged for rapid assessment of emerging threats and preparedness for future pandemics. NWSS was intentionally designed around health departments leading and coordinating programs as the end users of the wastewater data and formed communities of practice for public health implementers, laboratories, and wastewater utilities to better coordinate efforts nationally. For NWSS, health departments submit wastewater and associated metadata to CDC through the NWSS DCIPHER portal. CDC analyzes the data in real time and reports results to the health department for use in their COVID-19 response. As of June 2021, 31 states, 3 local, and 2 territorial public health departments are using CDC funds to support wastewater surveillance activities totaling ~$223M since the launch of NWSS in September of 2020. DCIPHER started accepting data into the system in January of 2021, and as of June 2021, the NWSS DCIPHER portal has received data from a little over 9800 unique wastewater samples.
